# Shape programming of liquid crystal elastomers by two-stage wavelength-selective photopolymerization

**DOI:** 10.1039/d5mh01907a

**Published:** 2025-10-28

**Authors:** Tom Bruining, Daniela R. Tomé, Danqing Liu

**Affiliations:** a Human Interactive Materials, Department of Chemical Engineering and Chemistry, Eindhoven University of Technology Groene Loper 3 5612AE Eindhoven The Netherlands danqing.liu@tue.nl

## Abstract

Reversible shape memory polymers are a topic of great interest in research focusing on materials for soft robotics and haptic technologies. In particular, liquid crystal elastomers (LCEs) programmed by two-stage crosslinking procedures have been increasingly reported in the past decade. These methods often include a base-catalyzed first crosslinking step, which limits the processibility of the material. Here, a two-stage crosslinking procedure based on the orthogonal wavelength-selective photo-initiation of free-radical and cationic ring-opening polymerizations is reported. Using a bifunctional acrylate-oxetane crosslinker, thermally responsive LCEs capable of reversible actuation are produced. The improved processibility of this method compared to base-catalyzed procedures is demonstrated through lithography-based production of actuators, as well as 4D-printed actuators with an additional shape programming step. The reported method opens up new manufacturing possibilities for the development of complex shape-programmed materials that were unattainable through previously known procedures.

## Abstract 2

We report a method to produce two-way shape memory liquid crystal elastomers using the orthogonal photo-crosslinking of acrylate and oxetane groups.

## Graphical abstract

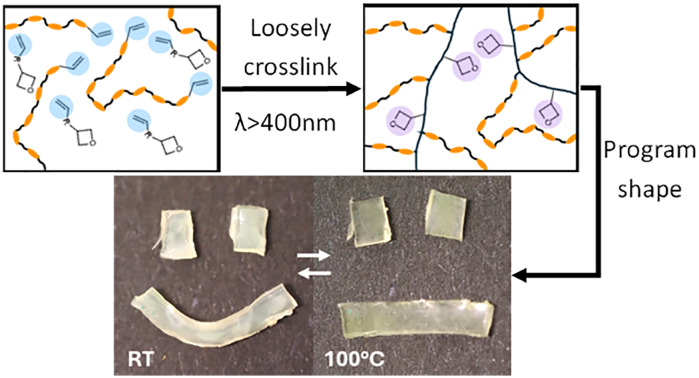

**New concepts**

In this research, we demonstrate a method for the production of liquid crystal elastomer actuators based on two-stage wavelength-selective photopolymerization. Previously reported two step crosslinking methods rely on a base-catalysed thiol-acrylate ‘click’ reaction to partially crosslink the liquid crystal polydomain, followed by a deformation and an additional polymerization step to program the material. The base-catalysed nature of this first step leads to issues with processing and upscaling. To address this limitation, we have created an innovative two-stage crosslinking approach that utilizes wavelength-selective photo-initiation of both free-radical and cationic ring-opening polymerizations. By photo-initiating both crosslinking steps, we gain spatiotemporal control over the first step, increasing the amount of available processing methods. As a proof of concept, we demonstrate the enhanced processibility by fabricating 4D-printed actuators as well as actuators fabricated with a photomask, both of which undergo a previously unreported additional shape programming step. This method opens up new possibilities for the production of more intricate shape-programmed actuators.

## Introduction

First proposed in 1975 by P. G. de Gennes,^1^ liquid crystal elastomers (LCEs) are a class of smart polymers consisting of an elastomeric network in which self-organizing mesogenic groups are incorporated. When subjected to an external stimulus, LCEs can exhibit outstanding mechanical and optical reversible actuation.^2–4^ These properties have incentivized researchers to explore the use of LCEs in applications such as mechanical actuators and sensors,^5,6^ soft robotics,^7–9^ haptics,^10–12^ and optics,^13^ among others. To obtain these actuations, the mesogens must be aligned along a common director. Strategies to program this alignment in liquid-crystalline polymers include surface anchoring, electric or magnetic fields, and shear flow.^14^

For the production of large, free-standing actuators, the most commonly used methods rely on a multi-step process. A first reaction step is used to form a randomly ordered, loosely crosslinked network, which is then mechanically deformed to induce alignment, followed by a second crosslinking step to permanently program the monodomain LCE.^15–18^ A thiol-acrylate “click” reaction is generally used to partially crosslink the LC polydomain into a gel-like state in its first step. A photopolymerization reaction using the remaining unreacted groups is then performed to stabilize the monodomain. In some cases, dynamic bonds are used to program the monodomain.^19–21^ Nevertheless, this base-catalysed first step has its drawbacks. As soon as the catalyst is introduced to the reaction mixture, it starts a crosslinking reaction, limiting the available time to manoeuvre and coat the mixture before the first crosslinking step is completed. This makes the method incompatible with coating techniques that require the mixtures to be stable for extended periods before coating. Moreover, if the reaction rate exceeds the solvent evaporation rate, solvent trapping could lead to irregularities in the LCE film.

An almost unexplored approach is to avoid this base catalysed crosslinking by using orthogonal photopolymerization reactions for both crosslinking steps. Dong *et al.* reported a method where a radical thiol–ene reaction under UV-light was followed by thermal curing step under visible light using a photo-base.^22^ In our research, we explored a two-stage wavelength-selective photopolymerization (TWSP) method based on acrylate and oxetane chemistry to produce actuators using relatively fast polymerization times under mild conditions. We also further explore and demonstrate unique processing steps that become possible using the improved spatiotemporal control from the orthogonal photo-initiated reactions.

**Fig. 1:**
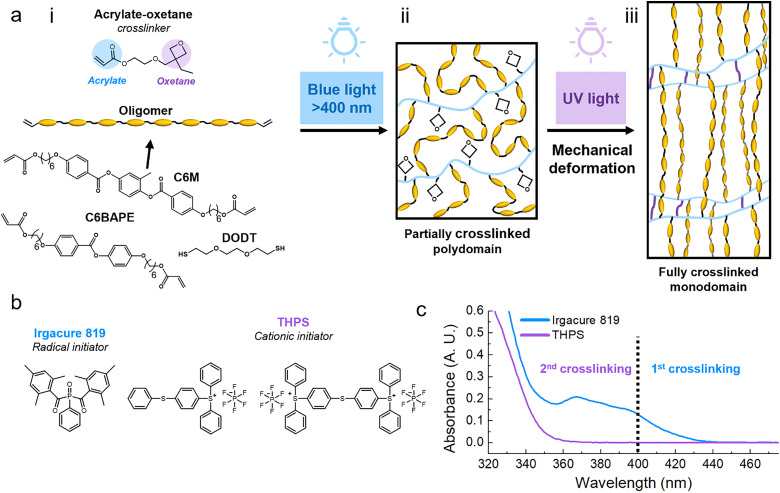
(a) Schematic illustration of the steps of the TWSP method for synthesizing and programming thermo-responsive LCEs: (i) overview of used monomers (ii) loosely crosslinked polydomain LCE produced in the first crosslinking step (iii) fully crosslinked LCE with mechanically programmed alignment. (b) Photoinitiators used in the production of shape memory polymers. (c) UV-vis absorption spectra of the photoinitiators Irgacure 819 and THPS, showing a non-overlapping region above 365 nm.

The orthogonal photopolymerization of acrylate and oxetane groups has been reported by El-Ghayoury *et al.* in a monomer containing both an acrylate and an oxetane functional group.^23^ Hoekstra *et al.* used a combination of liquid crystal diacrylate and dioxetane monomers to create interpenetrating LC networks, in which the orthogonality of the reactions was used to create a phase separated coating.^24,25^ In this work, we use the acrylate-oxetane monomer proposed by El-Ghayoury *et al.* as a crosslinker in a system with diacrylate LC oligomers to create shape-programmed actuators with two-way shape memory (Fig. 1a). This procedure makes use of two photo-initiators, Irgacure 819 and triarylsulfonium hexafluorophosphate salts (THPS) (Fig. 1b) that have a non-overlapping region in their absorption spectra (Fig. 1c). This allows for the initiation of a free-radical polymerization of acrylate groups using blue light (400–450 nm), creating a loosely crosslinked network with unreacted oxetane groups (Fig. 1aii). Using a mechanical deformation step, alignment can be programmed into this material. Using UV-light (320–400 nm), a cationic ring opening reaction can then be initiated to form additional crosslinks by polymerizing the oxetane groups (Fig. 1aiii). In the end, a permanently programmed monodomain LCE film capable of reversible “hands-free” actuation under thermal stimulus is obtained.

## Results and discussion

**Fig. 2:**
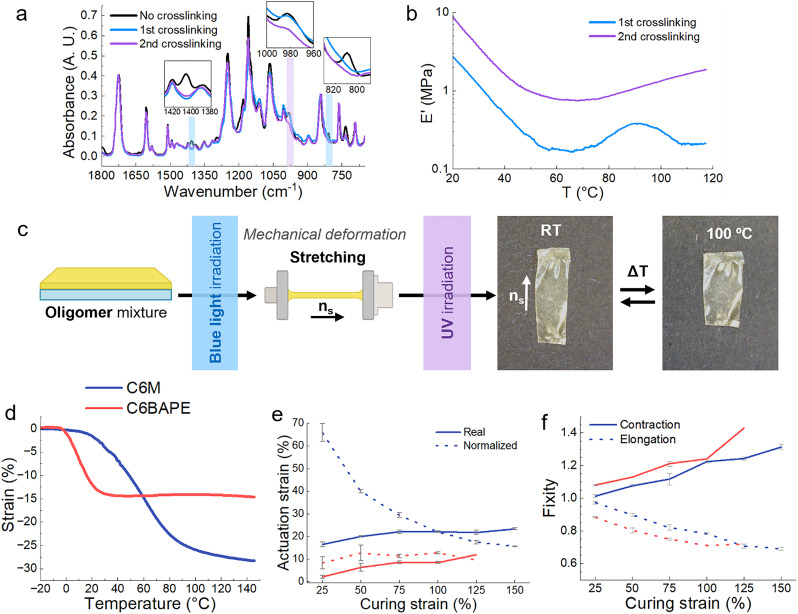
(a) Infrared absorption spectra before crosslinking, after the first crosslinking (partially crosslinked), and after the second crosslinking (fully crosslinked). The conversion of acrylates and oxetanes can be seen through the comparative decrease in intensity of their characteristic peaks, highlighted in blue and purple, respectively. (b) The storage modulus (*E*′) of the material in its rubbery state after first and second crosslinking. An increase in *E*′ is observed, confirming an increase in crosslink density after the second crosslinking. (c) Schematic representation of the production method of stretched shape memory actuators, resulting in a contraction-based actuation. (d) Strain variation percentage as a function of the temperature of C6M and C6BAPE samples produced with a curing strain of 100%. (e) Actuation strain and normalized actuation strain as function of curing strain in C6M and C6BAPE based samples. (f) Contraction and elongation fixities as a function of curing strain in C6M and C6BAPE based samples.

Two-way shape memory polymers were produced with two reactive mesogens (RMs), C6M and C6BAPE (Fig. 1ai). The differences between these mesogens originate primarily from their number of benzene rings. While C6M contains three benzene rings in its core, C6BAPE is comprised of only two. This weakens the intermolecular π–π interactions for the C6BAPE mesogen compared to C6M, promoting a decrease in onset transition temperatures.^26^ The actuators were fabricated by first synthesizing oligomers *via* base-catalysed thiol-acrylate Michael addition between the diacrylate RM, C6M or C6BAPE, and DODT (Fig. 1ai). A molar ratio of 1 : 0.9 diacrylate to dithiol was used to obtain an oligomer with a degree of polymerization (DP) of around 10 (the detailed procedure and characterization of the oligomers are specified in the supplementary information (SI), Fig. S4–S7 and Table S1). These oligomers were dissolved in dichloromethane (DCM) with the acrylate-oxetane crosslinker (synthesis specified in SI, Fig. S1–S3) and the two photo-initiators. The mixture was then coated onto a glass substrate. After evaporating solvent, the material was exposed to blue light to perform the first crosslinking step *via* free-radical polymerization (*λ* > 400 nm, 20 mW cm^−2^, 5 min). This step was performed at 60 °C, which is above the monomer mixture's nematic–isotropic transition temperature (*T*_NI_), to increase the mobility of the polymer chains, facilitating a high conversion in the free-radical polymerization step. The material was then cooled below *T*_NI_ to establish the alignment using a mechanical deformation. The second crosslinking step was then performed using UV light (320 < *λ* < 400 nm, 30 mW cm^−2^, 0–10 °C, 10 min) to initiate the cationic ring-opening polymerization step. Fourier-transform infrared spectroscopy (FT-IR) analysis showed the separate conversion of the acrylate and oxetane groups after each crosslinking stage (Fig. 2a). Characteristic acrylate peaks were found at ∼1410 cm^−1^ and ∼810 cm^−1^, related to the bending bands of the C–H bond in-plane and out-of-plane, respectively.^27^ These peaks disappear after the first crosslinking step with blue light, suggesting a nearly full conversion of acrylate groups. At ∼985 cm^−1^, the characteristic oxetane peak related to the C–O–C bond can be seen.^23^ This peak remains visible after the first crosslinking step, but significantly decreases in intensity after UV illumination. Thus, confirming that the cationic ring-opening photopolymerization occurred independently from the free-radical photopolymerization. The conversion rates during both crosslinking steps were determined through kinetics FT-IR (Fig. S8). Due to the low peak intensity, the signal to noise ratio was low, giving a good indication of the reaction times, but unreliable conversion data. The acrylates reached their maximum conversion after 1 min of blue light irradiation, Fig. 2a shows that the acrylate peaks fully disappear, indicating a full conversion. It took about 3 min under UV exposure to reach the maximum oxetane conversion of approximately 26%. This low oxetane conversion is likely caused by the limited mobility resulting from the network formed in the first reaction step. The formation of additional crosslinks in the second step was further confirmed by measuring the storage moduli (*E*′) of the material containing C6M before and after the UV-curing step using dynamic mechanical analysis (DMA) (Fig. 2b). After the second crosslinking, a significant increase in *E*′ is observed in the rubbery region of the material, which has a glass transition temperature (*T*_g_) around 0 °C (Fig. S9).

To produce functional actuators, molecular alignment needs to be established by performing a mechanical deformation between crosslinking steps. To test the actuation capability of the material, loosely crosslinked films were uniaxially stretched to induce alignment along the stretching direction (*n*_s_) (Fig. 2c). The second crosslinking step was then performed to permanently program the deformation. Polarized optical microscopy (POM) was used to confirm that the alignment was programmed by the second crosslinking step (Fig. S10). Finally, materials were obtained that contract along *n*_s_ and expand perpendicular to it when heated (Fig. 2c).

Stretched films were made analogously using the C6M and C6BAPE oligomers. To compare the thermal response of these materials, samples produced with 100% curing strain were characterized using DMA. A controlled force temperature ramp measurement was carried out to record their strain variation as a function of temperature (Fig. 2d). This shows a few clear differences in the effect of each mesogen on the actuation. The C6BAPE based material actuates at a lower temperature and in a stricter temperature range, starting from around 0 °C up to 30 °C. Whereas the C6M based material mainly actuates between 15 °C and 90 °C, but the temperature dependence levels off much slower, keeping some response up to the measurement limit of 150 °C. The total strain achieved by the materials also differs significantly, the C6M sample reaches a final 28% actuation strain, while the C6BAPE sample reaches only 14.5%. To verify the repeatability of the actuation, multiple actuation cycles were performed using a C6M sample, which showed no loss in actuation strain over several heating and cooling cycles (Fig. S11). This actuation is able to lift weights of at least 488 times the LCE film's own weight. A C6M sample weighing 27.7 mg with a starting length of 13 mm was able to lift a metal clip weighing 13.54 g by approximately 4.5 mm when heated (Fig. S12).

To more thoroughly explore the shape programming capabilities of these materials, samples were made at curing strains between 25% and 150%. Three samples were made for each data point, except at higher strains, where the samples were more prone to snapping. Only two C6M samples were obtained at 150%. For the C6BAPE, all samples stretched to 150% snapped and only one 125% sample was obtained. The curing strain (*ε*_c_), actuation strain (*ε*_a_), normalized actuation strain (*ε*_n_), elongation fixity (*f*_e_) and contraction fixity (*f*_c_) of these samples were determined based on the initial length before stretching (*l*_i_), the length of the sample while curing (*l*_c_), the final length (*l*_f_) of the fully cured sample, measured below *T*_NI_, and the actuated length (*l*_a_) of the sample at 100 °C. The full dataset is included in SI (Table S2). The following formulae were used for the calculations:
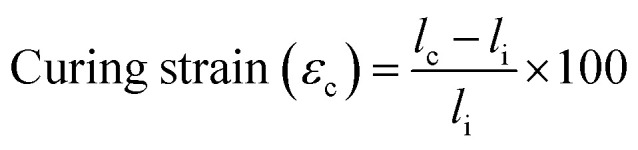

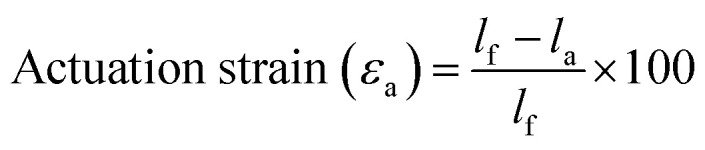

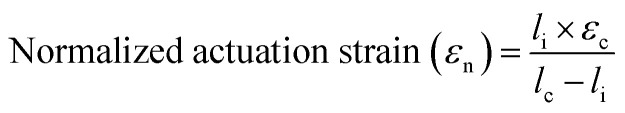

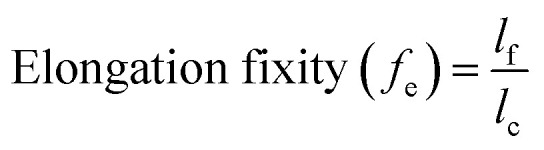

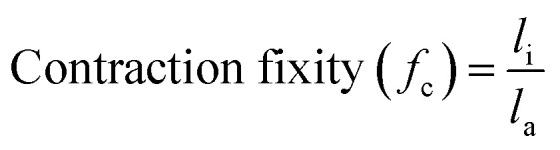


The actuation strains are plotted in Fig. 2e, the normalized actuation strains are included to give a better idea of the programming effectivity of the imposed curing strain. The elongation and contraction fixities are shown in Fig. 2f. The actuation strains at 100% curing strain in Fig. 2e are lower than those measured in Fig. 2d. We expect this is caused by the constant force applied in the DMA slightly stretching the samples, giving a higher length at low temperatures and raising the actuation strain.

As previously observed, the actuation strains of the samples containing C6BAPE are much lower than those of the samples containing C6M. The C6BAPE samples only reach around 10% actuation, while the C6M samples reach 25%. This difference is most likely related crosslink density after the first crosslinking step. We used oligomers with a similar degree of polymerization, however, since the C6BAPE mesogen has one less benzene ring compared to C6M, the molecular weight of the resulting oligomer is significantly lower (Fig. S4–S6 and Table S1). Resulting in a network with a lower molecular weight between crosslinks after the free-radical polymerization. Since the shape programming in systems with two crosslinking steps is dependent on the balance between the amount of crosslinking in the first and second step, this will have an influence on the obtained actuation and fixity.

In general, the obtained actuation strains increase with higher curing strain (Fig. 2e). However, the normalized actuation follows an opposite trend. The normalized actuation strain of the C6M samples starts around 65% at 25% curing strain, but drops when the curing strain is increased. The fixity values in Fig. 2f show a similar trend, at 25% curing strain, the C6M fixity values are close to 1. But for higher curing strains, the elongation and contraction fixity both deviate from this optimum value. For C6BAPE, the same trend is followed, but with values further from 1. This shows that in this composition, the material is more suited to program low curing strains, and the shape programming becomes less effective at higher strains, though these high strains do slightly increase the final actuation strain.

The order parameters (*S*) of the films were determined using wide-angle X-ray scattering (WAXS) (Fig. S13). The obtained diffraction patterns were interpreted using the Lovell and Mitchell method to estimate S.^28^ The order parameters were only measured for C6M samples, since the C6BAPE samples are isotropic at room temperature, and we could not measure them in a cooled state. A similar trend to the actuations and fixities was observed where the order parameter increases with increasing curing strain, but with a smaller relative increase. At 25% curing strain, *S* = 0.23, and S increases to 0.28 at 150% curing strain.

**Fig. 3:**
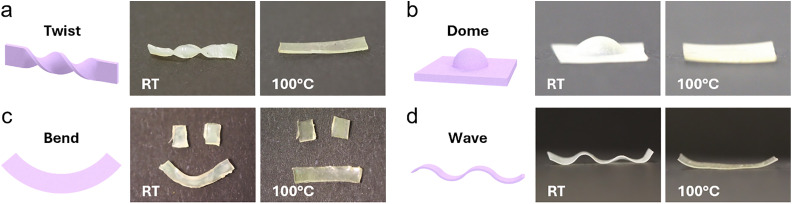
Actuation of shape-programmed elastomers produced with the TWSP procedure deformed by (a) twisting (680 μm film thickness), (b) stretching over a dome-shaped mould (260 μm film thickness), (c) bending (610 μm film thickness), and (d) bending back and forth to create a waved shape (230 μm film thickness).

The 3D shape programming capabilities of the material were further investigated by performing more complex deformations to induce alignment between the first and second crosslinking steps. The C6M based composition was used for these experiments as it produced the largest actuation strains. Fig. 3 depicts samples that were deformed by twisting, bending, and stretching over a mould. The material maintained the programmed shapes in its room temperature state and deformed back to its flat state when heated (videos of actuation in SI). For deformations that impose a low strain on the material, such as bending over a large radius or twisting, thicker samples were required to achieve successful shape programming than for deformations that impose more strain, like stretching over a mould or bending over smaller radii. This observation is in line with the findings of Barnes *et al.*^18^

**Fig. 4:**
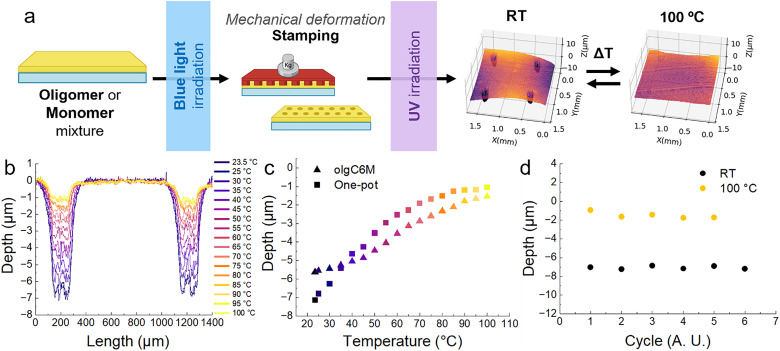
(a) Schematic representation of the production method of embossed shape memory actuators, resulting in an actuation behavior that switches between an indented state and a flat state. (b) 2D profile of the cross-section of two indents in the one-pot dots sample heated in 5 °C steps, showing the actuation decreasing the depth of the indents. (c) Depth of the indents in the olgC6M dots sample (169 μm thickness) and the one-pot dots sample (51 μm thickness) plotted over temperature. (d) Depth of indents in the olgC6M dots sample over multiple cycles of heating to 100 °C and cooling back down to room temperature.

To further advance the developed TWSP procedure, we propose an alternative in which the synthesis of the shape memory polymer does not require an oligomerization step. This improves the processability by reducing the complexity of the material preparation. In this case, pure reactive mesogen (C6M in Fig. 1a) was used in the first crosslinking. The necessary flexibility of the network was created by adding DODT (Fig. 1a), which acts as a chain transfer agent in the free radical polymerization. This decreases the crosslink density in the formed network, lowering the modulus of the material. Materials with various molar ratios of C6M : DODT, ranging from 1 : 0.9 to 0.7 : 1, were produced. For all ratios tested, the resulting material was too brittle to be stretched without snapping. This is likely caused by the different molecular structure created in the free-radical polymerization step compared to an oligomer- based material. With a higher ratio of thiols, the number of dangling ends in the network increases.^29^ The amount of long polymer chains in the mixture that would provide elasticity is thereby reduced, resulting in a more fragile material. Therefore, a different deformation that required less elasticity was used to instil alignment. To create shape memory actuation in this material, an embossing method was used. A stamp with protruding pillars was pressed into the loosely crosslinked LCE to create indents in the coating (Fig. 4a). These indents were then permanently programmed in the second crosslinking step. When heated, these indents deform to their original flat state, cooling back down reforms the indents’ depth. It was found that films containing higher concentrations of C6M, *e.g.*, 1 : 0.9 and 1 : 1 C6M : DODT, tended to crystallize around the edges of the film. Those films also relaxed back to their original configuration much faster once the stamp was removed, making it very difficult to perform the second crosslinking step to fix their shape. When the amount of mesogen was reduced, and the chain transfer agent was increased in the mixture, *e.g.*, 0.9 : 1 and 0.8 : 1 C6M : DODT, as expected, less brittle and more flexible samples were obtained. However, for even lower concentrations of mesogen, shape programming was much less successful, resulting in samples with fewer programmed indents (Fig. S14). The most successful shape programming was obtained using samples with a 0.9 : 1 C6M : DODT ratio, which will be designated as the one-pot dots sample from here on out. To draw a direct comparison between approaches, a stamped sample was also produced using the oligomer-based method, designated as the olgC6M dots sample. The surface profile of the samples was characterized using interferometry. The one-pot dots sample contained indents approximately 7 μm deep at room temperature, which returned to a near-flat state when heated to 100 °C (Fig. 4b). The olgC6M dots sample showed comparable actuation, containing indents that were approximately 6 μm deep at room temperature and reduced to 1 μm at 100 °C (Fig. S15). Heating gradually from room temperature to 100 °C, with 5 °C intervals, showed that the actuation, similar to the stretched samples, was not sharp but gradual. Plotting the indents’ depth over temperature gives a better visualization of the temperature-dependent actuation range (Fig. 4c). Approximately 90% of both samples’ actuation spread across a 60 °C temperature window. Comparing the olgC6M dots sample to the one-pot dots sample, it is clear that the olgC6M's actuation is sharper, showing a similar actuation range to the stretched samples. The onset actuation temperature of the one-pot dots sample is below room temperature, making the full actuation range impossible to record on the available setup. However, a slightly larger actuation was obtained using the one-pot method. The olgC6M dots sample lost 72.7% of its original depth at room temperature when actuated, while the one-pot dots sample lost 85.4%. This implies the one-pot procedure promotes the fabrication of samples capable of larger actuation. The repeatability of the actuation was explored by performing several cycles of heating and cooling (Fig. 4d). Plotting the depth of the indent at room temperature and 100 °C over each cycle shows no significant deterioration in actuation performance. The one-pot system is shown to be effective for creating surfaces with dynamic topographies. However, more work is needed to further develop the mixture to provide more flexibility after the first crosslinking step to apply it to free-standing actuators. For now, we chose to continue with the oligomer-based approach for demonstrations of possible processing methods.

**Fig. 5:**
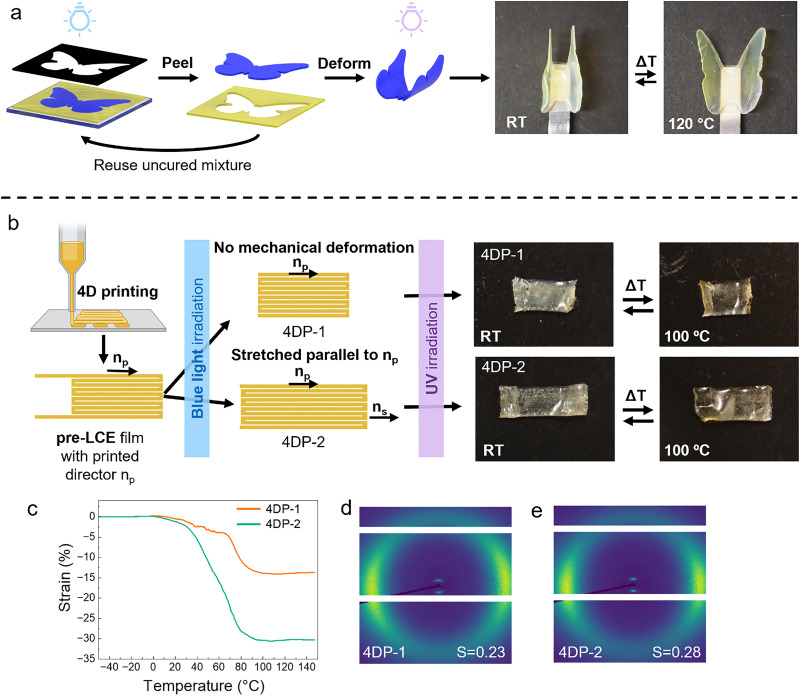
(a) The production of a shape memory actuator using a photomask with a butterfly-shaped cutout in the first crosslinking step to selectively cure regions of the monomer mixture. After the illumination step, the polymerized section was peeled from the glass substrate to obtain a loosely crosslinked butterfly shaped film. This was then bent and fully cured using UV-light to obtain a reversible actuator. (b) Schematic representation of the 4D printing procedure. Sample 4DP-1, was fully cured after printing without additional deformation steps. A second sample, 4DP-2, was cured after printing, stretched along the printing direction, and cured again. Both samples actuated by contracting when heated (c) actuation strain as a function of temperature for the 4DP-1 and 4DP-2 samples. (d) WAXS pattern of 4DP-1, corresponding to an order parameter of *S* = 0.23. (e) WAXS pattern of 4DP-2, corresponding to an order parameter of *S* = 0.28.

To demonstrate the spatiotemporal control granted by the photo-based first crosslinking step, we set out to make samples using a simple lithography step. As a proof of concept, we produced actuators using a photomask in the first illumination step (Fig. 5a). Coatings were produced using the olgC6M mixture analogously to the experiments described in Fig. 2. Before illuminating, a photomask with a cutout of a butterfly was placed directly on top of the coating. This protected part of the coating from the light used to cure the material, while keeping the rest exposed, selectively curing the material. To avoid effects of diffusion and obtain good curing selectivity, the samples were exposed to a high light intensity for a short time (*λ* > 400 nm, 70 mW cm^−2^, 4 s). After this, the illuminated section was sufficiently polymerized to be peeled from the glass substrate, separating it from the unreacted mixture. The resulting butterfly was then exposed to blue light for an additional minute to reach full conversion of the acrylates. Afterwards, it was bent and exposed to UV-light to perform the second crosslinking step as usual, resulting in a butterfly that flaps its wings in response to temperature. The unreacted mixture that was left over from making butterflies was reused to make more samples. After redissolving and coating the mixture, it was used to make caterpillar shaped actuators (Fig. S16). Though these shapes are simple, the results show that the material can be selectively cured and separated from the unreacted monomers while obtaining sharp edges. This could be exploited to create actuators with precise features, and could introduce a mechanical shape programming step to other lithography based techniques.

To give another demonstration of the benefit of the temporal control in the first crosslinking step, we explored the use of this material in direct ink writing. Direct ink writing of LCEs, also known as 4D printing, has been used in research to produce materials with complex shapes capable of different modes of responsiveness to the environment.^30^ In 4D printing, as in many other coating methods, the monomer or oligomer mixture must be stable inside a reservoir before being extruded. The mixture cannot contain an active catalyst as that would cause the material to crosslink inside the reservoir, preventing extrusion and clogging the device. This prevents the compatibility of most two-stage crosslinking materials with 4D printing. With our approach, materials can be printed and crosslinked as usual, containing alignment from the shear flow induced by the extrusion of the oligomer mixture. However, it allows for a second step where the material can be deformed to create additional alignment, followed by another crosslinking step. This sets it apart from current 4D printing literature, where alignment can exclusively be obtained from shear flow.

For the 4D printing experiments, the C6M oligomer-based monomer mixture was used. The material was successfully printed as a simple rectangular shape (10 × 4.88 mm, 180 μm thick) chosen for the experiment (Fig. 5b). After printing, the first crosslinking step using blue light was performed to fix the alignment induced by the extrusion of the material. The corresponding printed director is indicated as *n*_p_ in Fig. 5b. The first sample, 4DP-1, was fully cured without a deformation step before the second crosslinking with UV light. The second sample, 4DP-2 was stretched lengthwise, parallel to *n*_p_, with 140% strain before the second crosslinking step. The alignment obtained by the shear flow and mechanical programming of the obtained samples was confirmed using crossed polarizers (Fig. S17). A third sample, 4DP-3, was stretched perpendicular to *n*_p_ before being fully crosslinked. This was done as an exploratory step to examine the effect of stretching orthogonal to the printed alignments, this sample is discussed in SI (Fig. S18).

4DP-1 and 4DP-2 both actuate by contracting along the direction of the printing alignment and expanding perpendicular to it when heated (Fig. 5b). Thus, validating that the director instilled from printing is sufficient to obtain actuation. However, 4DP-2 shows a significantly larger actuation strain due to the additional stretching step used in its production. The actuation of the 4DP-1 and 4DP-2 samples was characterized through DMA controlled force temperature ramp measurements (Fig. 5c). 4DP-1 achieved 13.7% actuation strain, whereas 4DP-2 achieved over double the response, reaching 30.2% actuation strain. The order parameters of these samples were measured using WAXS. For 4DP-1, *S* = 0.23 (Fig. 5d) while for 4DP-2, *S* = 0.28 (Fig. 5e), showing that the stretching step improved the order parameter by about 20%.

## Conclusions

In summary, shape-programmable LCEs have been prepared using a two-stage crosslinking procedure based on the wavelength-selective photopolymerization of acrylate and oxetane functional groups. The use of photo-initiators with non-overlapping absorption bands in the blue light region allowed for the full separation of free-radical and cationic ring-opening polymerizations, used for the first and second crosslinking steps, respectively. A bifunctional acrylate-oxetane crosslinker was added to mixtures of commonly used LC monomers or oligomers to create reversibly actuating shape memory polymers. Free-standing actuators were produced using an oligomer-based procedure. Stretched samples were produced at different curing strains, revealing that the method is most effective at lower strains. Furthermore, actuators with more complex deformations, bending, twisting and stretching over a mould, were created. A one-pot, monomer-based procedure turned out to be unsuitable to produce free-standing actuators, but could be used to produce coatings with dynamic surface profiles. The added spatiotemporal control in the first crosslinking step granted by the catalyst-free nature of the TWSP approach was demonstrated using lithography and 4D-printing procedures. This shows the potential use of this material in bottom-up manufacturing procedures. Overall, this procedure expands the available processing options compared to other two-step crosslinking methodologies. In future research, this will allow for the production of more intricate initial states, opening up new possibilities to create complex actuators. Thereby promoting advancements in the fields of soft robotics and human-interactive materials.

## Author contributions

Tom Bruining and Daniela R. Tomé (conceptualization; data curation; formal analysis; investigation; methodology; validation; visualization; writing – original draft; and writing – review and editing); Danqing Liu (conceptualization; funding acquisition; project administration; supervision; and writing – review and editing).

## Conflicts of interest

There are no conflicts to declare.

## Supplementary Material

MH-013-D5MH01907A-s001

MH-013-D5MH01907A-s002

MH-013-D5MH01907A-s003

## Data Availability

The data supporting this article have been included as part of the supplementary information (SI). Supplementary information is available. See DOI: https://doi.org/10.1039/d5mh01907a.
