# Correction: Shape programming of liquid crystal elastomers by two-stage wavelength-selective photopolymerization

**DOI:** 10.1039/d5mh90140e

**Published:** 2025-11-18

**Authors:** Tom Bruining, Daniela R. Tomé, Danqing Liu

**Affiliations:** a Human Interactive Materials, Department of Chemical Engineering and Chemistry, Eindhoven University of Technology Groene Loper 3 5612AE Eindhoven The Netherlands danqing.liu@tue.nl

## Abstract

Correction for ‘Shape programming of liquid crystal elastomers by two-stage wavelength-selective photopolymerization’ by Tom Bruining *et al.*, *Mater. Horiz.*, 2025, https://doi.org/10.1039/D5MH01907A.

The authors regret an error in the formula for “contraction fixity” in the published article. The corrected formula is as follows:
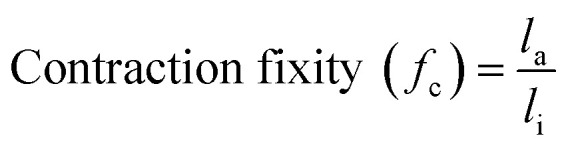


The Royal Society of Chemistry apologises for these errors and any consequent inconvenience to authors and readers.

