# Supplementary material for: Shape programming of liquid crystal elastomers by two-stage wavelength-selective photopolymerization
Source: Mater Horiz. 2025 Oct 28;13(1):243–51. doi: 10.1039/d5mh01907a (PMC12582633; doi:10.1039/d5mh01907a)
Supplement: MH-013-D5MH01907A-s001 [file MH-013-D5MH01907A-s001.pdf]

Supplementary Information (SI) for Materials Horizons.
This journal is © The Royal Society of Chemistry 2025

Supporting Information

Shape Programming of Liquid Crystal Elastomers by Two-stage Wavelength-selective Photopolymerization

Tom Bruining, Daniela R. Tomé, and Danqing Liu*

Table of Contents

Materials and methods 2
Coating Preparation 3
Oligomer-based Reaction Mixture 3
One-pot Reaction Mixture 3
Supplemental Experiments & Analysis 4
Synthesis of the Acrylate-oxetane Crosslinker 4
Oligomer Synthesis and characterization 6
Oligomer synthesis 6
NMR Analysis of the Oligomers 6
GPC Analysis of the Oligomers 8
DSC Analysis of the Oligomers 9
Kinetics FTIR: Acrylate & Oxetane Conversion 10
DMTA before and after 2nd crosslink 11
Stretched C6M and C6BAPE samples 12
Alignment 12
Actuation cycles 12
Weightlifting 13
Raw data for actuation and fixity calculations 13
XRD Measurements 14
One-pot Procedure 15
Interferometry Measurements of the Oligomer-based Stamped Sample 16
Alignment 16
4DP-3 17
References 17

Materials and methods

LC mesogen 1,4-Bis[4-(6-acryloyloxyhexyloxy)benzoyloxy]-2-methylbenzene (C6M) was purchased from Synthon GMBH. LC mesogen 4-(6-(acryloyloxy)hexyloxy)phenyl-4-(6-(acryloyloxy)hexyloxy)benzoate (C6BAPE) was purchased from Daken Chemical Limited. 2,2′-(Ethylenedioxy)diethanethiol (DODT, 95%), triarylsulfonium hexafluorophosphate salts (50wt.% in propylene carbonate), acryloyl chloride (97%) and triethylamine (TEA) were purchased from Merck Life Science NV. 1,8-diazabicyclo(5.4.0)undec-7-ene (DBU) was purchased from TCI Europe NV. Dichloromethane (DCM) was purchased from Biosolve B.V. Phenyl bis(2,4,6-trimethylbenzoyl)-phosphine oxide (Irgacure 819) was purchased from Ciba Specialty Chemicals NV. Magnesium sulfate (97%) was purchased from Fischer Scientific.

The degree of polymerization (DP), *i.e.,* average chain length, of the oligomers, as well as their number-averaged molecular weight (Mn), were determined from the proton nuclear magnetic resonance (^{1}H-NMR) spectra measured on a 400 MHz Bruker Advance III HD spectrometer. For these measurements, each oligomer was diluted in deuterated chloroform (99.8 atom% D). The data was processed using Mnova software. Gel permeation chromatography (GPC) measurements with a Shimadzu UV-3102 PC apparatus were executed to obtain the polydispersity index (PDI) of the oligomers. For this, tetrahydrofuran (THF) as the solvent and polydopamine (PDA) at 254 nm (with 4 nm intervals) as the detector were used. The phase transition temperatures of the oligomers were obtained via differential scanning calorimetry (DSC) measurements in TA instruments Discovery DSC 2500. The temperature ranged from - 50 to 150 °C with heating and cooling rates of 3 °C/min. Ultraviolet-visible (UV-vis) absorption spectra of the initiators (100 ppm concentration in ethanol) were extracted by a PerkinElmer Lambda 750 spectrophotometer with a 150 mm integrated sphere detector. The absorption measurements were taken between 150-550 nm with 1 nm intervals, and a response of 0.20 s by the detector. To determine the conversion of acrylates and oxetanes, Fourier-transform infrared spectroscopy (FT-IR) measurements were performed in a Varian 670-IR spectrometer. This data was interpreted via the Varian Resolution 4.0 software. To estimate the order parameter (S), X-ray diffraction (XRD) measurements were executed on a Ganesha lab instrument equipped with a Genix-Cu ultralow divergence source that generates X-ray photons with a wavelength and flux of 0.154 nm and 1×108 photons s^{-1}, respectively. Diffraction patterns were obtained using a Pilatus 300 K silicon pixel detector with 487×619 pixels of 172×172 μm^2. Silver behenate was used as a calibration standard. The sample-to-detector distance was 89 mm for wide-angle (WAXS) configuration. To study the stretched LCE films' mechanical properties as well as the degree and temperature range of the actuation, dynamic mechanical thermal analysis (DMA) measurements were executed in TA instruments Q800 DMA. To study the surface topography changes under temperature variations of the stamped samples, interferometry measurements via a Sensofar S Neox were carried out. With a Leica DM6000 polarized optical microscope (POM), the molecules' alignment in the LCE films could be determined. Note that all images were taken with the sample between cross-polarisers. A Hyrel 3D printer was used for the Direct Ink Writing of the LCEs, also known as 4D-printing of LCEs. Beforehand, the Autodesk Inventor 3D CAD software was used to make the three-dimensional (3D) design of the shape intended for printing. Then, this design was processed by the PrusaSlice software, which gave the basic Gcode for the programming of the printer machine. However, prior to and during the printing process, changes to the code were made. The nozzle used possessed a diameter of 400 μm, and the spacing between the printed lines was 1 μm. A good degree of reproducibility between printed rectangles was achieved.

Coating Preparation

Oligomer-based Reaction Mixture

To make the reaction mixture, 71.4 wt. % of the synthesized oligomer was combined with 17.9 wt. % acrylate-oxetane crosslinker, 3.6 wt. % Irgacure 819 and 7.1 wt. % triarylsulfonium hexafluorophosphate salts (THPS) in propylene carbonate (50 wt. % solution). The mixture was dissolved in DCM, stirred for ~ 2h, and then spread onto the substrate, after which the solvent was left to evaporate overnight at room temperature.

One-pot Reaction Mixture

For the one-pot approach, C6M was directly combined with DODT in various molar ratios, *i.e.,* 1:0.9, 1:1, 0.9:1, 0.8:1, and 0.7:1 (C6M:DODT), in the mixture. Except for these changing amounts of C6M and DODT the percentages of the other compounds in the mixture remained the same, 17.9 wt. % of acrylate-oxetane, 3.6 wt. % Irgacure 819, and 7.1 wt. % THPS, dissolved in DCM.

Supplemental Experiments & Analysis

Synthesis of the Acrylate-oxetane Crosslinker

The synthesis of the acrylate-oxetane crosslinker followed the procedure of El-Ghayoury A. *et al.* [1]. Starting from 3-(Hydroxyethylmethoxy)-3-ethyloxetane (Figure S1), which was in stock in the laboratory from synthesis for past projects. The NMR spectrum of this starting product is shown in Figure S2, the peaks marked as 'c' show the intact oxetane rings. First, in a 100 mL flask placed in an ice bath and under an argon atmosphere, 1.5 g or 9.36 mmol of 3-(Hydroxyethylmethoxy)-3-ethyloxetane were dissolved in 20 mL of DCM. To this solution 2 mL of triethylamine (TEA) catalyst was added. Next, 1.0 g or 0.89 ml or 11.04 mmol of acryloylchloride was combined in the mixture through the usage of a drop funnel. The resulting mixture was stirred for 30 min at 0 °C. Then, it was stirred overnight at room temperature. The following day, 200 mL of Water was added, and the compound was extracted with 3 x 100 mL of DCM. The organic layers were collected and dried over $MgSO_4$, filtered over silica, and the solvent was evaporated in a vacuum. Finally, the acrylate-oxetane crosslinker was obtained with yields around 80%. The NMR spectrum of the product is displayed in Figure S3, showing intact oxetane rings marked as 'c', acrylate groups marked as 'd'. The shift of the signals marked as 'a' and 'b' in figure S2 around 3.75 and 3.6 ppm respectively in the starting reactant to 4.35 and 3.75 ppm in the reaction product in figure S3 shows the successful synthesis of the acrylate-oxetane crosslinker.

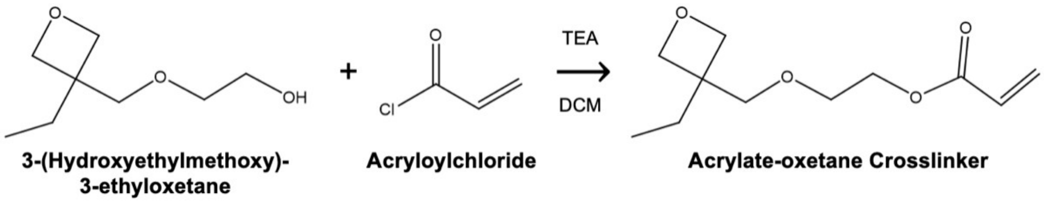

Figure S1. Chemical Reaction for the synthesis of the acrylate-oxetane crosslinker.

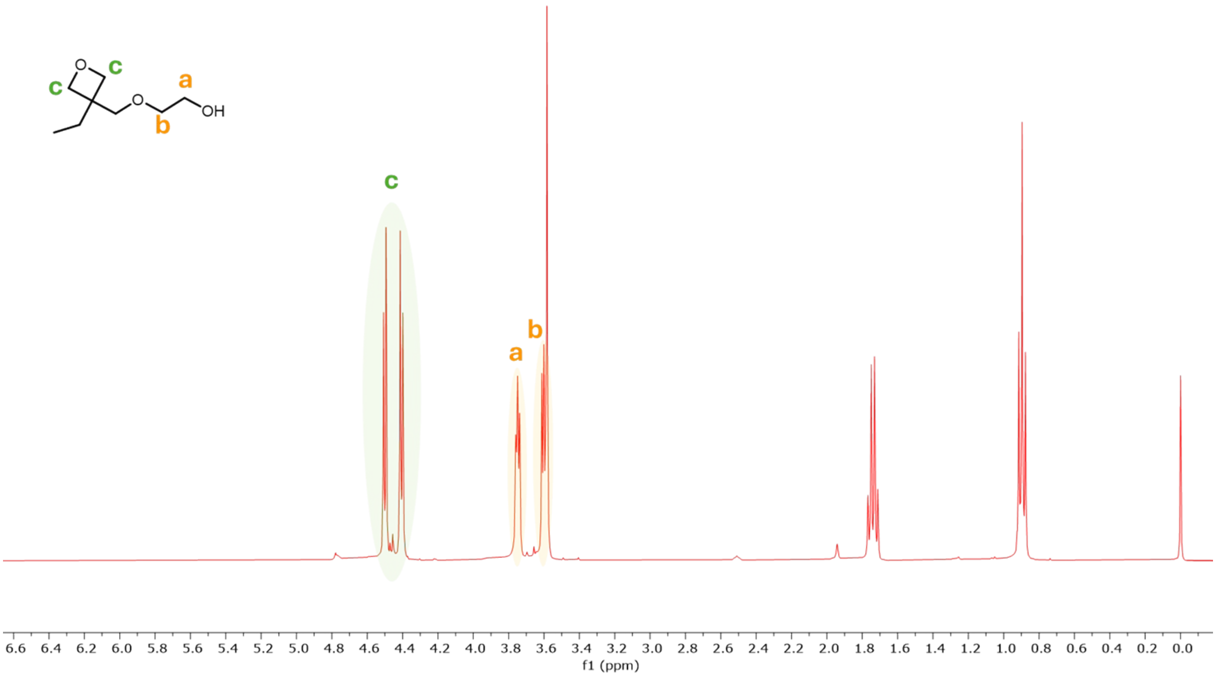

Figure S2. H-NMR spectrum of 3-(Hydroxyethylmethoxy)-3-ethyloxetane.

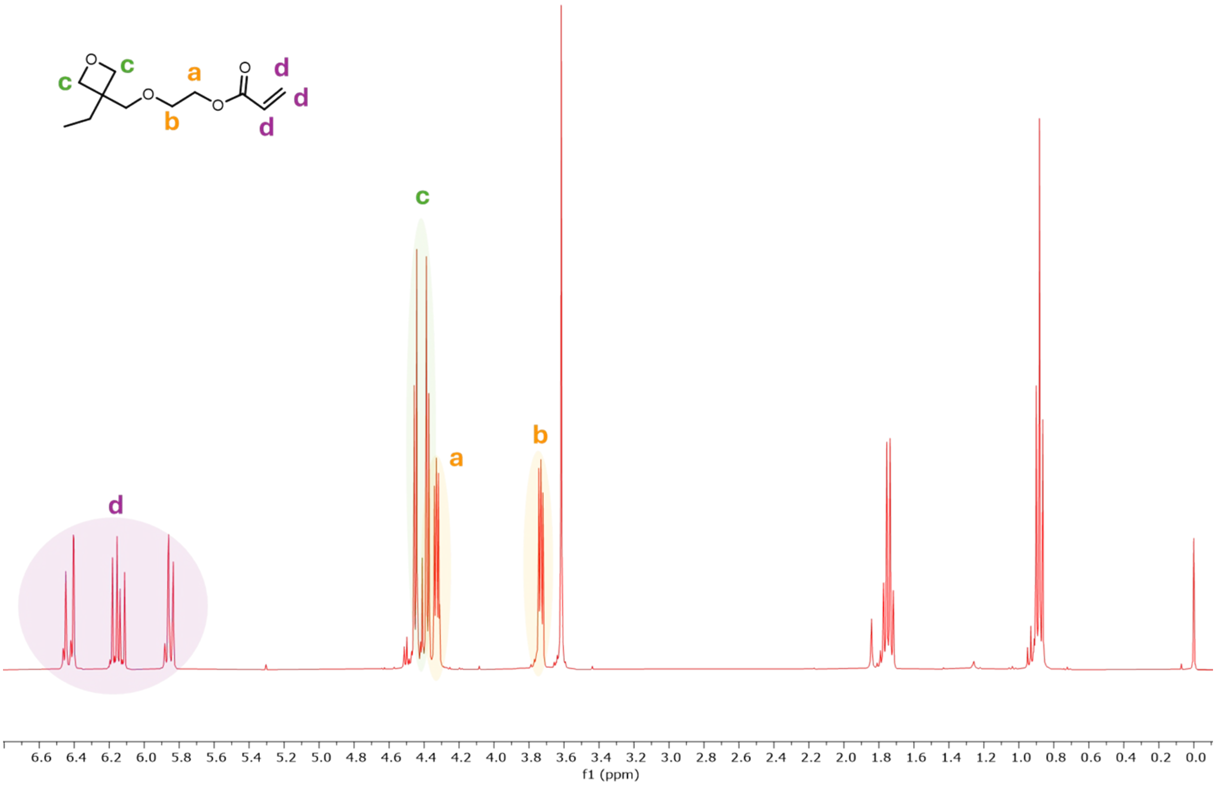

Figure S3. H-NMR spectrum of the synthesized acrylate-oxetane crosslinker 2-Propenoicacid,2-[(3-ethyl-3-oxetanyl)methoxy]ethylester.

Oligomer Synthesis and characterization

Oligomer synthesis

For the oligomerization step, two distinct types of mesogens were used to make the oligomers, for both, the LC mesogen : chain extender molar ratio was 1:0.9. The first mixture was comprised of 79.5 wt. % 1,4-Bis[4-(6-acryloyloxyhexyloxy)benzoyloxy]-2-methylbenzene (C6M) and 19.9 wt. % 2,2′-(Ethylenedioxy)diethanethiol (DODT). And the second 76.2 wt. % 4-(6-(acryloyloxy)hexyloxy)phenyl-4-(6-(acryloyloxy)hexyloxy)benzoate (C6BAPE) and 23.2 wt. % DODT. Note that in both, a 0.6 wt. % of 1,8-Diazabicyclo[5.4.0]undec-7-ene (DBU) was used as the base catalyst, and they were dissolved in dichloromethane (DCM). All aforementioned compounds were commercially obtained. Once all compounds were in solution, the mixture was stirred for ~ 2 h. Then, it was poured onto a dish for overnight solvent evaporation in a vacuum at room temperature.

NMR Analysis of the Oligomers

NMR measurements were performed to determine the DP, *i.e.,* average chain length, and Mn of the synthesized oligomers. The extracted spectra of the oligomers based on the C6M (olgC6M) and C6BAPE (olgC6BAPE) mesogens are shown in Figure S4 and Figure S5, respectively. The Mnova software was used to analyse the collected data.

In a C6M monomer, four aromatic protons in the mesogenic core correspond to the peak at 8.13 ppm (Figure S4 a). Whereas the acrylate groups on either side of the molecule appear in three NMR peaks at 6.41 (Figure S4 b), 6.12 (Figure S4 c) and 5.83 ppm (Figure S4 d), which each correspond to two protons. Therefore, in a C6M monomer, the ratio of the area of an acrylic peak to the aromatic peak is 4:2 or 2:1.

In an oligomer produced with a C6M to DODT ratio of 1:0.9, the ends of the oligomer chains will consist of C6M moieties and therefore contain unreacted acrylate groups that provide the same NMR signals as the C6M monomer. As such, to calculate the DP, the integral of the aromatic peak at 8.13 ppm must be divided by twice the integral of one of the acrylic peaks. The DP and Mn of olgC6M were calculated as follows:

$$DP_{olgC6M} = \frac{24.26}{2} = 12.13 \approx 12\ repeating\ units\ (r.u.)$$

$$Mn_{olgC6M} = (Mn_{r.u.} \times DP) - M_{DODT} =$$
$$= [(672.77 + 182.30) \times 12.13] - 182.30 = 10\ 182.70\ g/mol$$

Where $Mn_{r.u.} = M_{C6M} + M_{DODT}$. Note that the need for the subtraction of the molecular mass of the DODT (M_{DODT}) comes from the fact that in the oligomer there is always one less DODT section compared to the number of C6M sections.

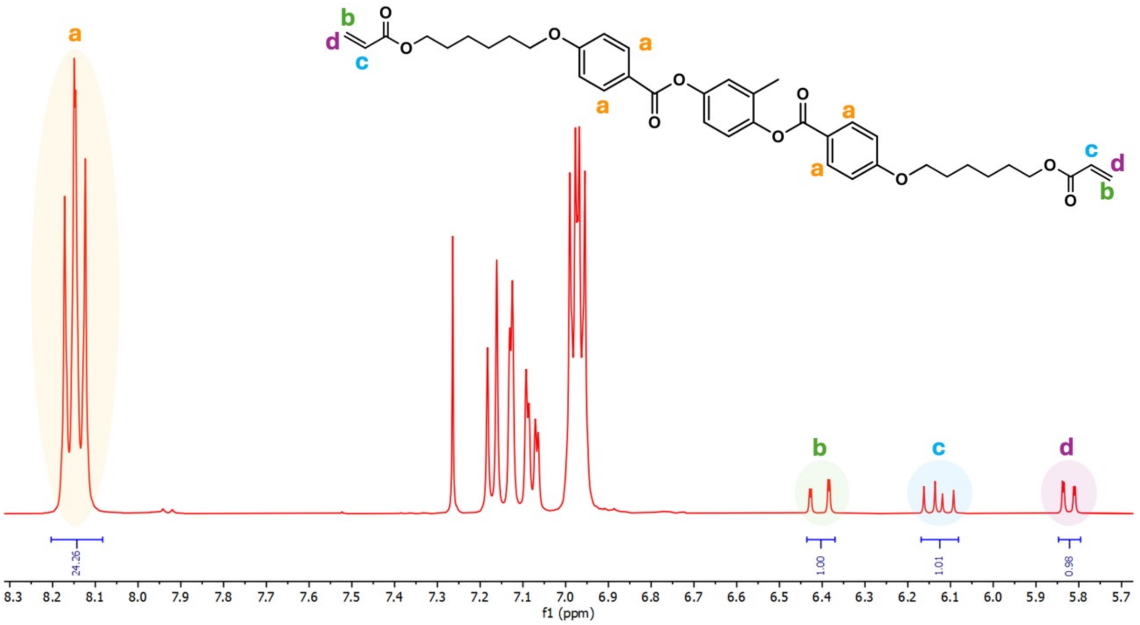

Figure S4. ^{1}H-NMR spectrum of olgC6M, where **a** relates to four aromatic protons of the mesogenic core, while **b**, **c**, and **d** each correspond to two protons of the acrylate groups at the two extremities of the monomer.

Contrary to the previous case where the LC mesogen was C6M, when it is C6BAPE instead, the DP corresponds directly to the integral of the aromatic protons peak when the acrylic peaks are normalized to 1, since the ratio in the monomer is two protons of the mesogenic core per two protons of the two acrylate groups (*i.e.,* 1:1 ratio). The remaining calculi are similar with just the molecular weight being different since the mesogen changed:

$$DP_{olgC6BAPE} = 10.81 \approx 11\ r.u.$$

$$Mn_{olgC6BAPE} = (Mn_{r.u.} \times DP) - M_{DODT} =$$
$$= [(538.64 + 182.30) \times 10.81] - 182.30 = 7\,611.06\ g/mol$$

Where $Mn_{r.u.} = M_{C6BAPE} + M_{DODT}$.

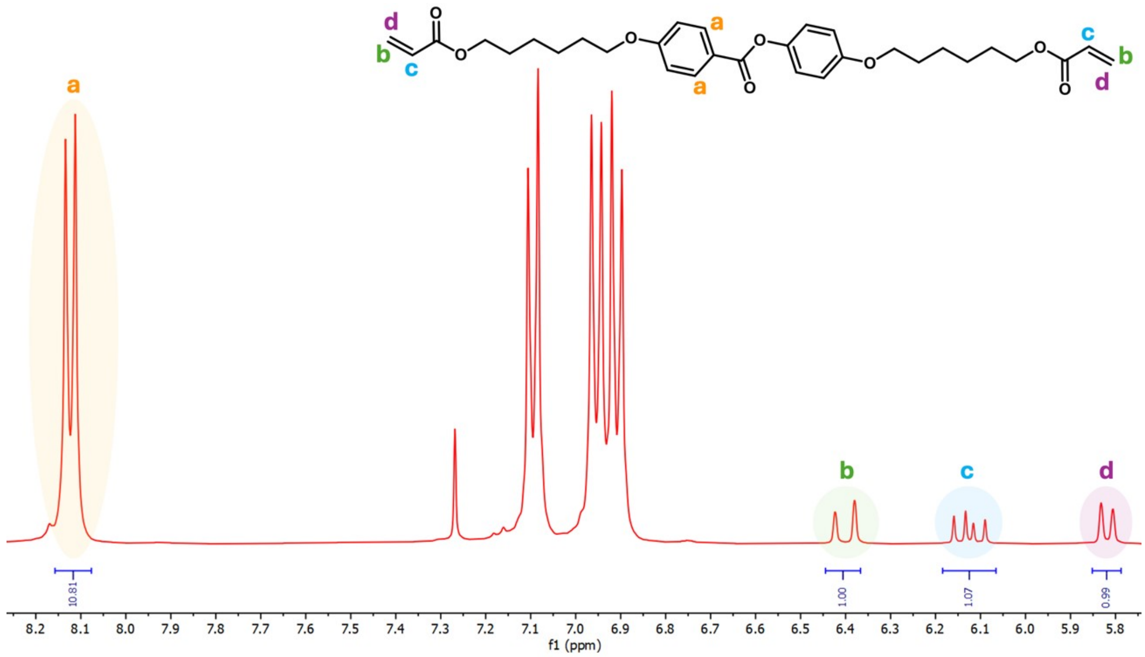

Figure S5. ^{1}H-NMR spectrum of olgC6BAPE, where **a** relates to two aromatic protons of the mesogenic core, while **b**, **c**, and **d** each correspond to two protons of the acrylate groups at the two extremities of the monomer.

GPC Analysis of the Oligomers

To determine the PDI, GPC measurements were effectuated. The different-sized mesogenic cores of C6M or C6BAPE were detected through their absorptions at a 254 nm wavelength. Note that the detector used was PDA. Figure S6 shows the normalized absorption as a function of time plot, made from the data extracted from the GPC measurement of each synthesized oligomer. The GPC results were also more neatly organized in table S 1 to facilitate interpretation. In it, the various molecular weights are presented. The fraction of the Mw as the numerator and the Mn as the denominator gives the value of the PDI of each oligomer.

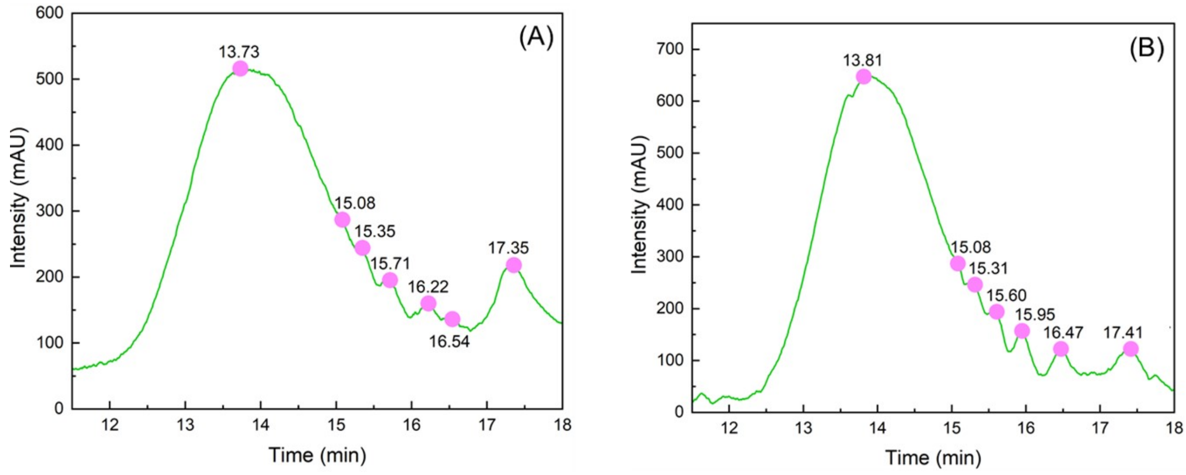

Figure S6. GPC plots of (A) olgC6M and (B) olgC6BAPE.

The oligomer with the C6BAPE mesogen showed a higher PDI than olgC6M as shown in **Table S 1**.

Table S 1. GPC analysis results for the three oligomers. Where Mn is the number average molecular weight, Mw is the weight average molecular weight, and Mz is Z average molecular weight.

Oligomer	Mn [g/mol]	Mw [g/mol]	Mz [g/mol]	PDI [A. U.]
olgC6M	13 944	33 382	53 220	2.39
olgC6BAPE	10 483	26 954	45 756	2.57

DSC Analysis of the Oligomers

To investigate the individual phase transitions of the oligomers, DSC measurements were performed. Knowing the T_{NI} of the material is crucial for the programming of the alignment. Figure S7 exhibits the DSC endothermal and exothermal curves of each oligomer, with the respective T_{SN}, and T_{NI} peaks marked.

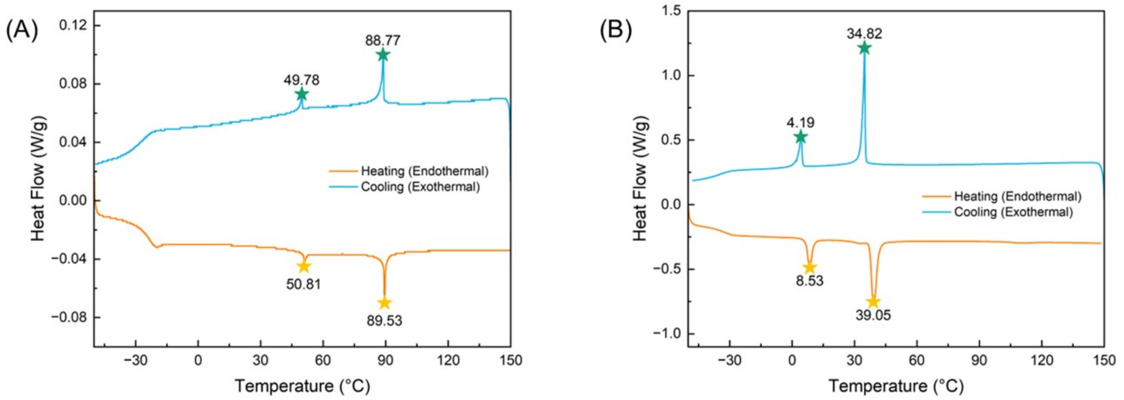

Figure S7. DSC Endothermal and Exothermal curves of (A) olgC6M and (B) olgC6BAPE.

Kinetics FTIR: Acrylate & Oxetane Conversion

With kinetics FTIR measurements, the decrease in intensity of the acrylate peak at 1410 cm^{-1} during the first crosslinking step (Figure S8 A) and of the oxetane peak at 985 cm^{-1} during the second crosslinking step (Figure S8 B) were determined. From this data the conversion of each of these chemical groups in their respective reaction steps were calculated. It should be noted that the measured data is very noisy. This is caused by the relatively low concentration of the reactive groups in the material, which makes the peaks corresponding to these groups appear very small in the FTIR spectrum, as is visible in Figure 1 d in the main text. Because of this, the peak height is not much larger than the noise in the baseline during the measurement, while also partially overlapping with other peaks. When using Varian Resolution 4.0 to determine the area of the peak to calculate the conversion, this causes a lot of noise, as is visible in Figure S8. To make the data possible to interpret, the raw data was first smoothed and subsequently fitted to determine the conversion values. Though this amount of noise and data processing makes the obtained conversion data unreliable but gives a good indication of the time required to reach the maximum attainable conversion.

In Figure S8 (A), exponential growth can be seen until around 1 min, and from then on, the curve goes down. Acrylates, once converted, cannot go back to being unreacted. Therefore, the exponential growth seen in the beginning corresponds to the conversion of acrylates, whereas the decrease in conversion cannot indicate an increase of unreacted acrylates. Instead, it is related to the analysis program picking up the signal from the two neighbouring peaks which partly overlap with the acrylate peak. However, looking at the spectrum before and after the 1st crosslinking step (Figure 1 d) shows that the acrylate peak fully disappears. Therefore, we can conclude from this measurement that the conversion of acrylates at 1410 cm^{-1} was at least 88% after about 1 min of blue light irradiation.

As for the curve of oxetane conversion, presented in Figure S8 (B), the fit chosen was exponential asymptotic since the decrease in intensity of this peak is exponential in the beginning (approximately the first 3 min) and then becomes constant. This result shows that, after ~ 3 min of UV irradiation, ~ 26% of oxetanes at 985 cm^{-1} were converted.

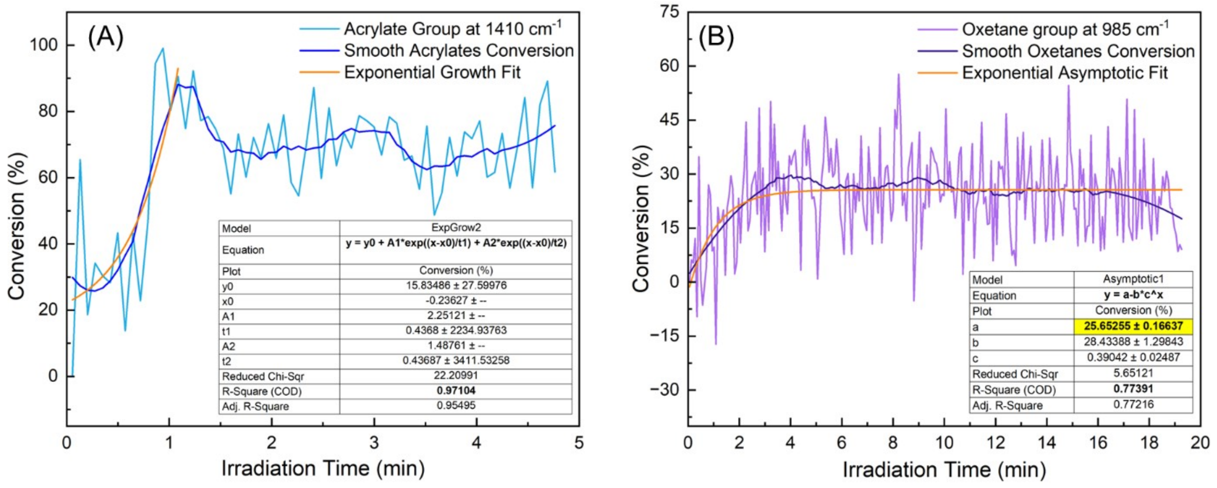

Figure S8. Conversion plots calculated from the intensity decrease over irradiation time determined through kinetics FTIR measurements: (A) acrylate group at 1410 cm^{-1} wavenumber (~ 88% conversion after ~ 1min); (B) oxetane group at 985 cm^{-1} wavenumber (~ 26% conversion after ~ 3min).

DMTA before and after 2nd crosslink

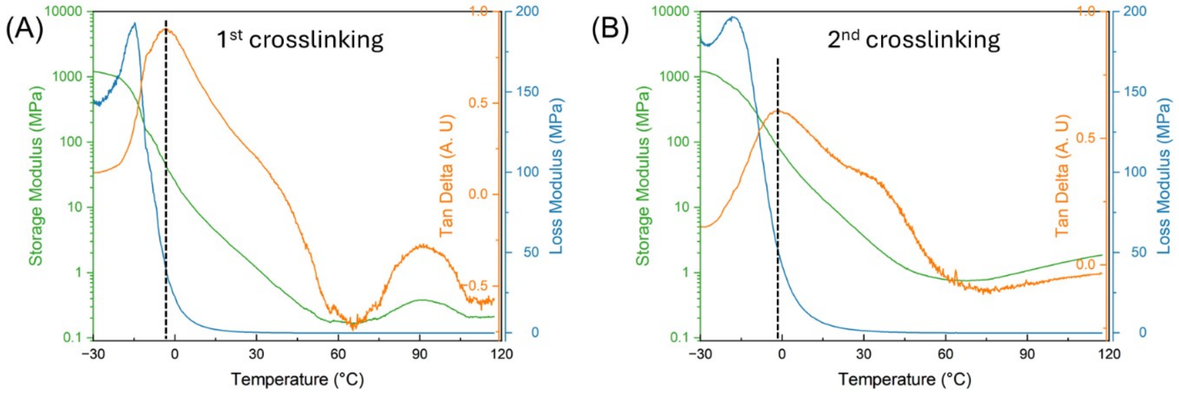

Figure S9. DMA measurements of a C6M sample A) after the first crosslinking step. B) After the second crosslinking step. Compared to the sample after one crosslinking step, the glass transition temperature was raised slightly, and the storage modulus in the rubbery region has increased after the second crosslinking.

Stretched C6M and C6BAPE samples

Alignment

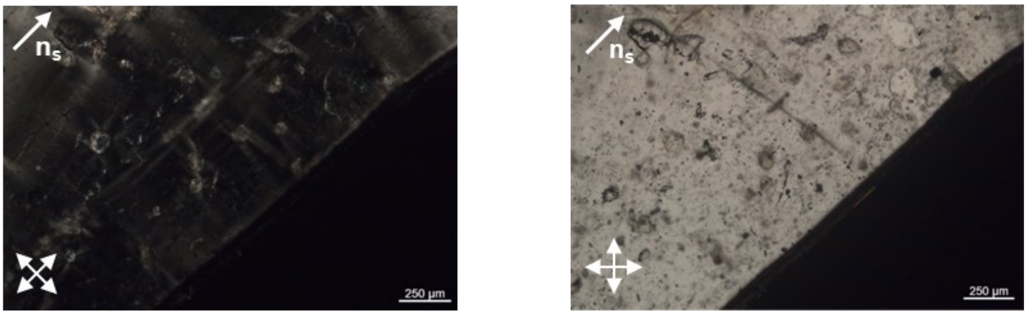

Figure S10. Stretched and fully crosslinked C6M sample as observed in a polarized optical microscope between crossed polarizers. When the polarizers are at 0 and 90° to the stretched direction n_s of the sample, a dark image is obtained. When the polarizers are oriented at 45° to n_s, the sample appears bright, while the background remains black. This demonstrates that an alignment was succesfully programmed.

Actuation cycles

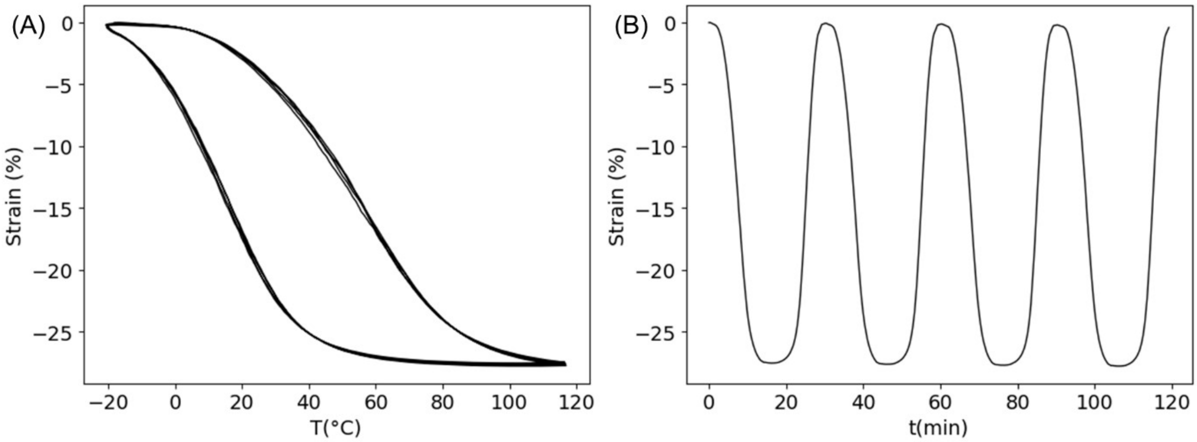

Figure S11. Actuation strain over four heating and cooling cycles measured using DMTA in constant force mode with a heating/cooling rate of 10 °C/min. (A) Plotted over temperature to show excellent repeatability, and (B) plotted over time to clearly show the separate cycles.

Weightlifting

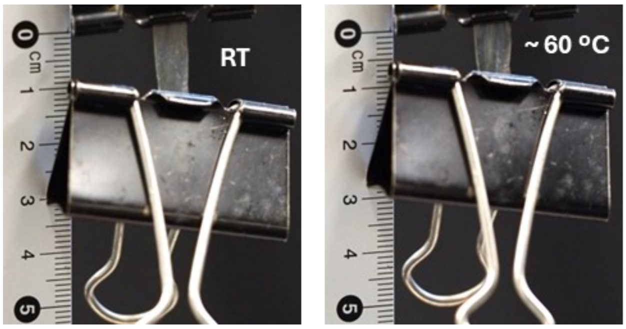

Figure S12. C6M sample weighing 27.7 mg with a starting length of 13 mm lifting a metal clip weighing 13.54 g by approximately 4.5 mm upon heating with a heat gun

Raw data for actuation and fixity calculations

Table S 2. Initial length (l_i), curing length (l_c), final length (l_f) below T_{NI} and actuated length (l_a) of C6M and C6BAPE based stretched samples. All given lengths are in mm.

		C6M		C6BAPE	
l_i	l_c	l_f (10 °C)	l_a (100 °C)	l_f (0 °C)	l_a (100 °C)
16	20	19.7	16.2	17.6	17.3
16	20	19.3	16	17.6	17.4
16	20	19.2	16.4	17.7	17.1
16	24	21.2	17.0	19.2	18.1
16	24	21.6	17.4	18.8	18.1
16	24	21.8	17.3	19.9	18.0
16	28	22.0	16.8	21.1	19.5
16	28	22.9	18.0	20.8	18.8
16	28	23.7	18.7	21.4	19.6
16	32	25.2	19.6	22.7	19.9
16	32	25.4	19.7	22.4	21.1
16	32	24.7	19.4	22.8	19.8
16	36	26.4	20.1	25.9	22.8
16	36	24.8	19.7		
16	36	25.2	19.9		
16	40	27.0	20.8		
16	40	27.8	21.2		

XRD Measurements

The X-ray scattering pattern of an LC material allows for the qualitative and quantitative analysis of the mesophases present at a specific temperature. In this work, all XRD measurements were performed on the LCE films at RT.
First, observing the results shown in Figure S13 from a qualitative lens, two outer vertically symmetric half circumferences can be seen for the C6M sample. These are related to the order along the director, while the inner symmetric half circumferences emerge from the additional order orthogonal to the director, which is associated with a smectic phase. It must be noted that the straight white horizontal lines present in all of the samples, where nothing was measured, come from the equipment itself and not the samples.

From the diffraction patterns, the order parameters of the samples were estimated through the Lovell and Mitchell method detailed in the work of Mark T. Sims *et al.* [2]. For a more detailed explanation of this method, we take a C6M sample as an example (Figure S13 (A)). First, the intensity change as a function of the azimuthal angle variation was extracted from the X-ray scattering pattern. This plot, shown in Figure S13 (B), possesses two regions where the intensity values decrease significantly, and these correspond to the white lines seen in Figure S13 (A). Nevertheless, these error regions will not interfere with the estimation of the S, since only a quarter of a rotation (90º) is needed for the calculations. In this case, the region chosen was from 90º to 182º. Note instead of the number of points corresponding to exactly a 90º rotation, one or two additional points must be taken for correct calculations. These values are then inserted in the equation shown below to determine the S.

$$S = -2 \cdot \frac{1}{2}\left(3 \cdot \frac{\int_0^{\frac{\pi}{2}} I(\phi)\sin\phi(\cos\phi)^2 d\phi}{\int_0^{\frac{\pi}{2}} I(\phi)\sin\phi d\phi} - 1\right)$$

Where S is the order parameter, I is the intensity, and ϕ is the azimuthal angle. The order parameters for the olgC6BAPE, 4DP-1 and 4DP-2 samples were calculated analogously.

Finally, after calculating the order parameters for C6M samples produced with different curing strains, the results are plotted in Figure S13 C. This shows linear trend of increasing order parameters with increasing curing strains. At 25% curing strain, S is 0.23, and S increases to 0.28 at 150% curing strain. The relative increase in order parameters is much smaller than the increase in curing strain. Combined with the actuation and fixity data shown in Figure 2 of the main text, this confirms that at higher strains, the capability of the material to program the induced alignment decreases.

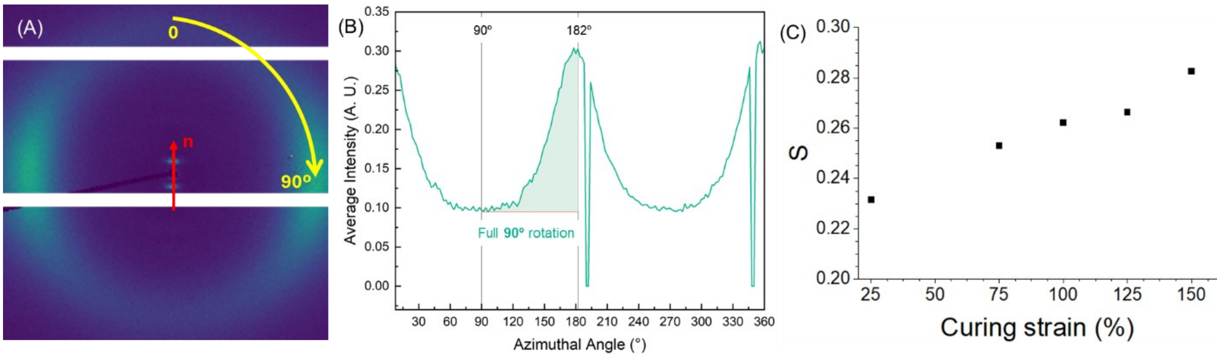

Figure S13. X-ray scattering pattern of (A) the olgC6M sample (B) olgC6BAPE sample, which is close to fully isotropic since its transition temperature is below room temperature. (C) Azimuthal profile of the intensity extracted from the olgC6M samples X- ray pattern shown in (A).

One-pot Procedure

To achieve samples of the highest quality through the one-pot approach, different C6M:DODT molar ratios were experimented with, namely 1:0.9, 1:1, 0.9:1, 0.8:1, and 0.7:1. Note that the concentrations of the other chemicals and the remaining TWSP procedure were kept the same and all samples were stamped with the same mould.

A surface examination of the individual films yields valuable insights into their structural characteristics. Figure S14 shows the films after stamping and fully crosslinking the samples, the successfully programmed dots are marked with coloured outlines. The films containing higher concentrations of mesogen (Figure S14 A and B) were much more brittle after fully crosslinked, showing crystallization at their edges. They also tended to relax back to their original configuration much faster, making it very difficult to cure them uniformly while keeping the programmed topography. When the amount of mesogen was reduced, and the chain extender was increased in the mixture, as expected, less brittle and more flexible samples were obtained (Figure S14 C and D). However, if the film was not made of an adequate amount of LC material, the shape memory reduced and less dots were successfully programmed (Figure S14 E).

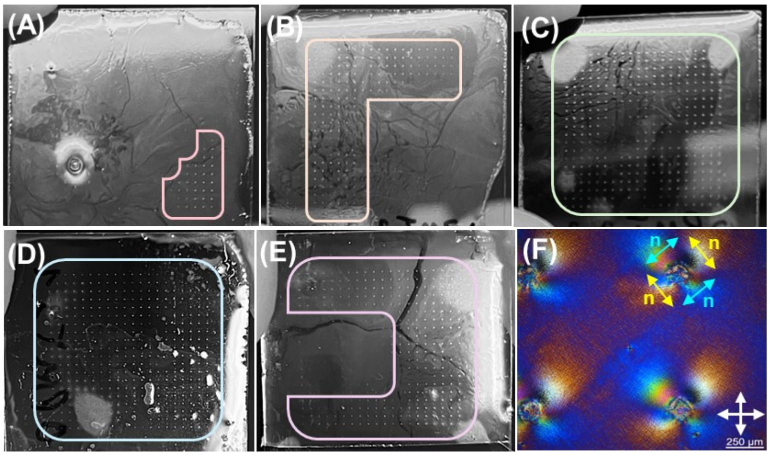

Figure S14. One-pot dots samples, with the C6M:DODT molar ratios: (A) 1:0.9; (B) 1:1; (C) 0.9:1; (D) 0.8:1; (E) 0.7:1. The regions with successful alignment programming (dots) are surrounded by different colored shapes (cooler

tones=more dots; warmer tones=less dots). (F) POM with a first-order retardation plate image, where the azimuthal alignment emerging from the center of each dot is seen on the one-pot (0.9:1 C6M:DODT molar ratio) dots sample.

Interferometry Measurements of the Oligomer-based Stamped Sample

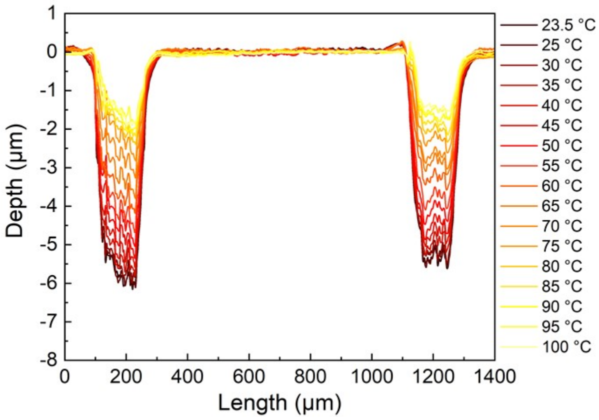

Figure S15. Depth *vs* Length plots over a temperature range from 23.5 to 100 ºC, with 5 ºC steps of olgC6M dots sample.

Lithography recycling

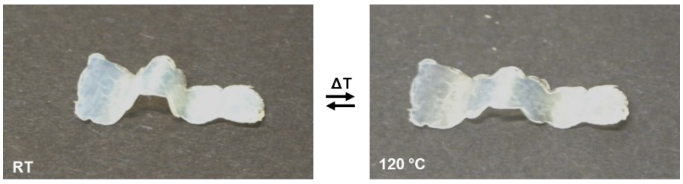

Figure S16. Caterpillar shaped actuators that were produced by recycling the leftover materials from the production of the butterfly shaped actuators from Figure 5 in the main text. The unexposed material was dissolved in DCM and coated on a glass plate. It was treated analogously to the lithography method described in the main text, but with a different photomask. The sample was bent at multiple spots after the 1st crosslinking to create a crawling-like actuation.

4D printing

Alignment

To investigate alignment resulting from 4D printing and an additional stretching step. The 4DP-1 and 4DP-2 samples were placed between cross-polarizers on top of an LED panel. As can be observed in Figure S17, both samples display a dark state when parallel to the polarizers and a bright state when positioned at 45° to the polarizers. This demonstrates the presence of a planar alignment in 4DP-1 and 4DP-2. Note that the brighter regions on the extremities of these samples in the dark states originate from the printing procedure. To print rectangles, the printhead needs to make a U-turn at the end of each line, in these turns, the director follows the printing path, resulting in a different alignment at the extremities of the sample compared to the bulk. The contrast between the bright and dark state of 4DP-2 is noticeably larger than that of 4DP-1, showing that an increase in order parameter is obtained through the stretching step.

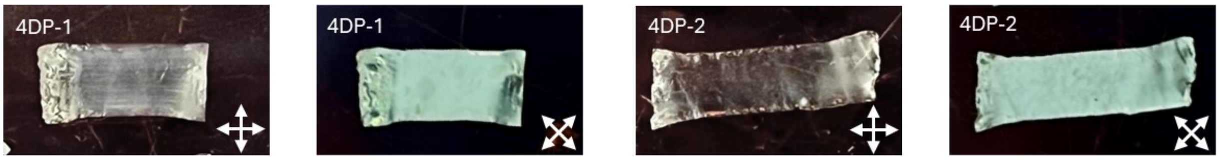

Figure S17. 4DP-1 and 4DP-2 between crossed polarizers on top of an LED panel.

4DP-3

As mentioned in the main text, 4DP-3 was made by stretching the printed sample perpendicular to n_p (Figure S18 A). The resulting alignment was observed between crossed polarizers (Figure S18 B). It exhibits a more peculiar alignment compared to the previous stretched samples. The centre appears dark in when n_p is oriented parallel to the polarizers and bright at 45°, but sections in the corners of the sample show the opposite behaviour. This indicates an induced rotation of the director from the edges to the centre of the sample. It seems that in the centre of the sample, the director follows n_s, while at the top and bottom edges, the alignment is oriented in between n_p and n_s.

4DP-3 actuated by contracting along the direction of stretching. However, no contraction or expansion along n_p was observed. This suggests there is competition between the two alignments. As actuation purely based on the printed alignment would cause a contraction along n_p and expansion perpendicular to it, as demonstrated with 4DP-1. For 4DP-3, actuation based exclusively on the alignment induced by stretching would cause an expansion along n_p. Thus, the material is programmed in ways that should induce both an expansion and a contraction along the same axis, resulting in a neutralized state with no actuation along np. Besides contracting, the material also bends when actuating (Figure S18 C), most likely due to some asymmetry induced in the stretching step.

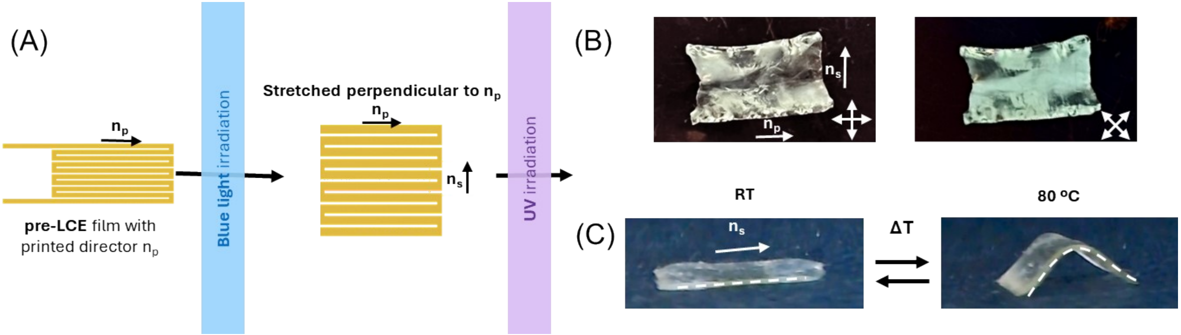

Figure S18. A) The schematic production of the 4DP-3 sample, which is stretched perpendicular to the printed alignment between both crosslinking steps. B) 4DP-3 observed between crossed polarizers on an LED panel. C) Actuation of 4DP-3 when heated to 80°C.

References

1. El-Ghayoury, A., Boukaftane, C., De Ruiter, B. & Van Der Linde, R. Ultraviolet-ultraviolet dual-cure process based on acrylate oxetane monomers. *J Polym Sci A Polym Chem* **41**, 469–475 (2003).
2. Sims, M. T., Abbott, L. C., Richardson, R. M., Goodby, J. W. & Moore, J. N. Considerations in the determination of orientational order parameters from X-ray scattering experiments. *Liq Cryst* **46**, 11–24 (2019).
